# YiQiFuMai injection for chronic heart failure: Protocol for a systematic review and meta-analysis

**DOI:** 10.1097/MD.0000000000009957

**Published:** 2018-02-23

**Authors:** Yuanping Wang, Xiaohui Li, Ziqing Li, Yu Zhang, Dawei Wang

**Affiliations:** a The Second Clinical College of Guangzhou University of Chinese Medicine, Guangzhou; b Shunde Hospital Affiliated of Guangzhou University of Chinese Medicine, Shunde, China.

**Keywords:** chronic heart failure, protocol, systematic review, YiQiFuMai injection

## Abstract

**Background:**

Chronic heart AQ4 failure (CHF) is the final stage of various heart diseases. YiQiFuMai injection (YQFMI) has been widely applied in the treatment of CHF. However, to our knowledge, there has been no systematic review or meta-analysis of randomized controlled trails (RCTs) regarding the effectiveness of this treatment. Here, we provide a protocol to evaluate the efficacy and safety of YQFMI for CHF.

**Methods:**

To evaluate the clinical efficacy of YQFMI in treating CHF, 2 researcher members will independently search the RCTs in the following 8 Chinese and English databases, in which the data collection will be from the time when the respective databases were established to January 2018. The databases will include MEDLINE, EMBASE, Cochrane CENTRAL, CINAHL, the Chinese Biomedical Literature Database, the China National Knowledge Infrastructure, VIP Information and Wanfang Data. The therapeutic effects according to the mortality and the New York Heart Association (NYHA) function classification will be accepted as the primary outcomes. We will use RevMan V.5.3 software as well to compute the data synthesis carefully when a meta-analysis is allowed.

**Results:**

This study will provide a high-quality synthesis of current evidence of YQFMI for CHF from several aspects including mortality, NYHA function classification.

**Conclusion:**

The conclusion of our systematic review will provide evidence to judge whether YQFMI is an effective intervention for CHF.

PROSPERO registration number: PROSPERO CRD42017079696.

## Abstract 2

Supplemental Digital Content is available in the text

## 1 Introduction

As a major public health issue, chronic heart failure (CHF) is the final stage of various heart diseases. Epidemiological studies have shown that the prevalence of CHF is about 1% to 2% in western countries with about every 5 to 10 in 1000 people diagnosed with the disease per year.^[1]^ More than 650,000 new CHF patients are added annually only in the United States.^[2]^ The disease is not only the leading cause of death, hospitalization, and rehospitalization, but also seriously affects the patients’ quality of life.^[3,4]^

Vasodilators, diuretics, and anti-hypertensive are the conventional treatment methods for CHF.^[5–7]^ According to a large number of randomized controlled trails (RCTs), the drugs can reduce the mortality of patients and effectively alleviate the symptoms.^[8]^ It has been proved that angiotensin receptor blockers (ARB), angiotensin-converting enzyme inhibitors (ACEi), aldosterone antagonists, and beta blockers can inhibit the progression of myocardial reconstruction and slow down the development of CHF.^[9]^ However, there are well-known adverse events that will limit the use of the traditional drugs. For instance, ACEI can cause adverse reactions such as allergic reactions, cough, and impairment of renal function.^[10]^ Meanwhile, headache, legs edema, bradycardia, and premature ventricular contraction are common adverse reactions associated with antihypertensive drugs.^[11,12]^ The use of diuretics by CHF patients tends to cause the electrolyte disturbance.^[11]^ In addition, combination of multiple medications increases the risk of adverse reactions in the patients.^[13,14]^ Therefore, it is essential to find a method that can treat CHF effectively with less side effects.

As a supplementary treatment, Chinese herbal medicine (CHM), which originated in ancient China and has long been based on traditional Chinese medicine (TCM), is used widely to treat cardiovascular diseases in China, such as CHF.^[15,16]^ YiQiFuMai injection (YQFMI) is a modern Chinese medical preparation that derives from TCM prescription Shengmai San, which consists of Radix Ginseng, Ophiopogonis Radix, and Schisandrae Chinensis Fructus.^[17,18]^ Animal experimental studies demonstrated that YQFMI could improve the cardiac function and significantly decrease the activity of the inflammatory mediators, such as tumor necrosis factor alpha and interleukin-6, in the mice with CHF.^[19,20]^ There is also another animal experiment report that NF-κB inactivation and cytokine suppression might be one of the main mechanisms of YQFMI that caused ameliorative effects in CHF mice.^[21]^ Many RCTs showed that the clinical outcome of using YQFMI together with western medicine was better than that of using western medicine alone for patients with CHF, with a lower mortality rate and less side effects.^[22,23]^ As far as we know, however, there has not been any meta-analysis study on the efficacy and safety of YQFMI in treating CHF yet. Hence, the main purpose of this study is to evaluate the efficacy and safety of YQFMI in treating CHF with systematic review of relevant clinical studies. In addition, the clinical trial scheme is to be improved by analyzing the current situation of YQFMI in the treatment of CHF clinical trials.

## 2 Methods

### 2.1 Inclusion criteria for study selection

#### 2.1.1 Types of patients

Based on the criteria established by the New York Heart Association (NYHA),^[24]^ all patients included in the study will be adults diagnosed with CHF, without limit on sex, age, and race. Animal studies will be excluded.

#### 2.1.2 Types of interventions

The patients included in the study will be treated with the traditional western medications and YQFMI in the treatment group according to the diagnosis and treatment guidelines of heart failure, while the control group will be treated with the traditional western medications.

#### 2.1.3 Types of outcome measures

##### 2.1.3.1 Primary outcomes

- Mortality
- NYHA function classification

##### 2.1.3.2 Secondary outcomes

- Quality of life as measured by various instrument
- Exercise test or 6-minute walk test performance
- Hospitalization and rehospitalization
- Adverse effects

### 2.2 Search methods for the identification of studies

To evaluate the clinical efficacy of YQFMI in treating CHF, 2 researcher members will independently search the RCTs in the following 8 Chinese and English databases, in which the data collection will be from the time when the respective databases were established to January 2018. The databases will include MEDLINE, EMBASE, Cochrane CENTRAL, CINAHL, the Chinese Biomedical Literature Database, the China National Knowledge Infrastructure, VIP Information and Wanfang Data. Medical keywords and uncontrolled terms will be combined to retrieve the data and the retrieval strategy will be decided after several preretrievals. Searched terms such as YQFMI, CHF, and RCT will be covered. Meanwhile, the references in the included trials and the original literature of the subject-related systematic evaluations will be attained as the supplementary literature to ensure the recall rate. Take PubMed as an example, see Appendix 1 for the retrieval strategy.

#### 2.2.1 Searching other resources

The research members will also manually retrieve the relevant literature, such as comprehensive/alternative and complementary medical textbooks and clinical guidelines for all related tests, and contact with the experts in the field and corresponding author to obtain the important information that cannot be available in the retrieval.

### 2.3 Data collection and analysis

#### 2.3.1 Selection of studies

**Figure 1:**
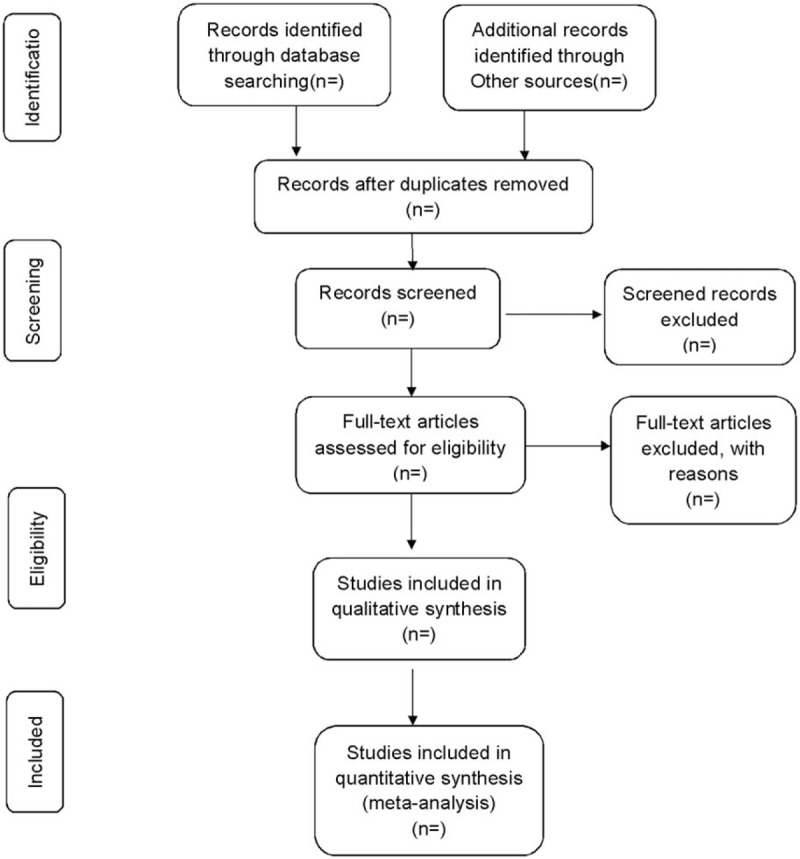
PRISMA flow chart. PRISMA = preferred reporting items for systematic review and meta-analysis.

Two reviewers will each read the titles and abstracts of the retrieved articles to rule out the obvious unrelated items in accordance with the inclusion criteria created beforehand. They will then read the whole text of the articles that meet the requirements, extract useful data and check the final inclusion of the references. In case of disagreement, the reviewers shall discuss with or consult a third research member. As for the information unavailable, the author of the original paper will be contacted. The process of studies selection is presented in a preferred reporting items for systematic review and meta-analysis (PRISMA) flow diagram (Fig. 1).

#### 2.3.2 Data extraction and management

Two researchers will collect data from the included literature, including but not limited to diagnosis, comorbidity, course and severity of disease, sample size, age, sex, intervention and the specific treatment adopted in the control group, follow-up, outcome indicators, results of the study and adverse events. Where there are missing data, errors, and ambiguity, the problems will be solved through group discussion, communication with the author, or third-party arbitration.

#### 2.3.3 Assessment of risk of bias in included studies

The Cochrane Collaboration's tool provided by Cochrane Handbook for Systematic Reviews of Interventions will be adopted by 2 trained research members to evaluate the risk of bias in randomized trials. The main items include random sequence generation, allocation concealment, blinding method for patients, researchers and outcomes assessors, incomplete result data, selective reports, and other potential problems. The results of the evaluation will be divided into level of low-risk, unclear, and high-risk. If there is any inconsistency, the problem will be resolved through group discussion, communication with the author to confirm details, and arbitration with a third-party.

#### 2.3.4 Measures of treatment effect

The relative risk (RR) will be used to indicate the enumeration data while the mean difference (MD) the measurement data, and the effect sizes will be presented with a 95% confidence interval (CI) for analysis.

#### 2.3.5 Dealing with missing data

The researchers will try to attain the missing data by contacting the corresponding author of the referenced paper. If that cannot work, we will build the analysis on available data.

#### 2.3.6 Assessment of heterogeneity

The heterogeneity of the results of the included studies will be analyzed through chi-square test (*α* = 0.1), with its value determined by *I*^2^. The statistic heterogeneity among trials can be ignored if *I*^2^ is ≤50%, and the effect size will be computed using the fixed effects model. If *I*^2^ >50%, the heterogeneity among the trials will be significant.

#### 2.3.7 Assessment of reporting bias

First of all, the visual asymmetry on a funnel plot will be used to determine whether a publication bias exists when >10 trials are included in the study. If the image is unclear, the research members will use STATA 11.0 software to conduct the quantitative analysis of the Egger test.

#### 2.3.8 Data synthesis

The software RevMan 5.3 (The Cochrane Collaboration, Oxford, England) will used for meta-analysis. If there is no statistic heterogeneity among the results of the included studies, the analysis will be performed with the fixed effects model. On the other hand, the cause of the heterogeneity should be further analyzed if a statistical heterogeneity is found. The random effect model will be employed to carry out the analysis with the effect of the obvious clinical heterogeneity is excluded. When obvious clinical heterogeneity is observed, the researchers can turn to the subgroup or sensitivity analysis, or only descriptive analysis.

#### 2.3.9 Subgroup analysis

If a relatively obvious heterogeneity is discovered in the included studies, the researchers will conduct a subgroup analysis based on the patients’ age, sex, CHF type, and treatment period.

#### 2.3.10 Sensitivity analysis

A sensitivity analysis of the main outcome indicators will be conducted to determine the robustness of the results if there are enough included trials. The researchers will re-evaluate the main outcome indicators to decide whether these factors affect the results of meta-analysis, with the low-quality literature and studies of small sample size excluded.

#### 2.3.11 Ethics and dissemination

The results of the systematic review will be disseminated via publication in a peer-reviewed journal and presented at a relevant conference. The data we will use do not include individual patient data, so ethical approval is not required.

## 3 Discussion

The incidence of CHF increases year by year with a large aging population in the world.^[9]^ Therefore, the search for a safe and effective drug or non-drug therapy has become a much-talked-about and received widespread attention from the global medical community. The existing clinical studies have indicated that YQFMI can effectively improve the clinical symptoms of patients with CHF, such as fatigue and shortness of breath, and has fewer side effects.^[22,23]^ However, the exact mechanism remains to be further explored. There has not been any meta-analysis of the clinical efficacy and safety of YQFMI in treating CHF in English. Our research will be divided into 4 parts: identification of studies, selection of studies, data analysis, and data extraction and management. We hope that this study can provide more rigorous medical evidence for the efficacy and safety of YQFMI in treating CHF. However, there may be some potential deficiencies in this study. For instance, different doses and courses of treatment in the intervention group in the included trials may lead to relatively significant heterogeneity in the meta-analysis results. In addition, it may result in a certain bias since the databases from which this study retrieves literature does not include those in South Korea and Japan.

Preferred reporting items for systematic review and meta-analysis protocols (PRISMA-P) checklist of this protocol is presented in online supplementary.

## Supplementary Material

Supplemental Digital Content
