# Supplementary material for: YiQiFuMai injection for chronic heart failure: Protocol for a systematic review and meta-analysis
Source: Medicine (Baltimore). 2018 Feb 23;97(8):e9957. doi: 10.1097/MD.0000000000009957 (PMC5842014; doi:10.1097/MD.0000000000009957)
Supplement: Supplemental Digital Content [file medi-97-e9957-s001.doc]

**Appendix 1. Search strategy used in PubMed database**

#1 Cardiac Failure OR Heart Decompensation OR Decompensation, Heart OR Heart Failure, Right-Sided OR Heart Failure, Right Sided OR Right-Sided Heart Failure OR Right Sided Heart Failure OR Myocardial Failure OR Congestive Heart Failure OR Heart Failure, Congestive OR Heart Failure, Left-Sided OR Heart Failure, Left Sided OR Left-Sided Heart Failure OR Left Sided Heart Failure

#2 YiQiFuMai OR [Yi-Qi-Fu-Mai](https://www.ncbi.nlm.nih.gov/pubmed/27473956)

#3 Randomized controlled trial OR clinical study OR Clin-ical Trial OR Controlled study OR Controlled Trial OR Random*Control* study OR random* Control* Trial

#1 AND #2 AND #3
